# Deep learning predicts malignancy and metastasis of solid pulmonary nodules from CT scans

**DOI:** 10.3389/fmed.2023.1145846

**Published:** 2023-05-19

**Authors:** Junhao Mu, Kaiming Kuang, Min Ao, Weiyi Li, Haiyun Dai, Zubin Ouyang, Jingyu Li, Jing Huang, Shuliang Guo, Jiancheng Yang, Li Yang

**Affiliations:** ^1^Department of Respiratory and Critical Care Medicine, The First Affiliated Hospital of Chongqing Medical University, Chongqing, China; ^2^Dianei Technology, Shanghai, China; ^3^University of California, San Diego, San Diego, CA, United States; ^4^Department of Radiology, The First Affiliated Hospital of Chongqing Medical University, Chongqing, China; ^5^School of Computer Science, Wuhan University, Wuhan, China; ^6^Shanghai Jiao Tong University, Shanghai, China; ^7^École Polytechnique Fédérale de Lausanne, Lausanne, Switzerland

**Keywords:** deep learning, malignancy, metastasis, solid pulmonary nodule, CT

## Abstract

In the clinic, it is difficult to distinguish the malignancy and aggressiveness of solid pulmonary nodules (PNs). Incorrect assessments may lead to delayed diagnosis and an increased risk of complications. We developed and validated a deep learning-based model for the prediction of malignancy as well as local or distant metastasis in solid PNs based on CT images of primary lesions during initial diagnosis. In this study, we reviewed the data from multiple patients with solid PNs at our institution from 1 January 2019 to 30 April 2022. The patients were divided into three groups: benign, Ia-stage lung cancer, and T1-stage lung cancer with metastasis. Each cohort was further split into training and testing groups. The deep learning system predicted the malignancy and metastasis status of solid PNs based on CT images, and then we compared the malignancy prediction results among four different levels of clinicians. Experiments confirmed that human–computer collaboration can further enhance diagnostic accuracy. We made a held-out testing set of 134 cases, with 689 cases in total. Our convolutional neural network model reached an area under the ROC (AUC) of 80.37% for malignancy prediction and an AUC of 86.44% for metastasis prediction. In observer studies involving four clinicians, the proposed deep learning method outperformed a junior respiratory clinician and a 5-year respiratory clinician by considerable margins; it was on par with a senior respiratory clinician and was only slightly inferior to a senior radiologist. Our human–computer collaboration experiment showed that by simply adding binary human diagnosis into model prediction probabilities, model AUC scores improved to 81.80–88.70% when combined with three out of four clinicians. In summary, the deep learning method can accurately diagnose the malignancy of solid PNs, improve its performance when collaborating with human experts, predict local or distant metastasis in patients with T1-stage lung cancer, and facilitate the application of precision medicine.

## 1. Introduction

Lung cancer is the leading cause of cancer-related death worldwide (1–3). Pulmonary nodules (PNs) are an early and potentially curable form of lung cancer (4). In screening for lung cancer, the average detection rate of PNs has increased to 22.00%−59.70%, of which < 5% are malignant nodules (5–7). Early diagnosis of malignant solid nodules is especially important to improve the prognosis of lung cancer due to its indeterminate aggressive characteristics (8–11). Although several studies have shown that the rate of malignancy in solid nodules is lower than that in ground-glass nodules, distinguishing benign and malignant solid PNs is even more difficult than distinguishing ground-glass nodules due to their overlapping characteristics with lung cancer in CT imaging (12–16). It was reported that among excision PNs, the proportion of benign lesions can be as high as 51.67%, and most of them were solid nodules (17, 18). Meanwhile, metastasis accounts for a vast majority of lung cancer-related deaths (19). Early screening for lung cancer has shown an increased detection rate of early-stage lung cancer, with some small nodules having been found to have metastases at preliminary diagnosis (10, 11, 20–22). Accurate TNM staging is an important prerequisite for the treatment of lung cancer. At least 20% of patients who undergo curative lung surgery relapse with undiagnosed metastatic disease, indicating that the current approach, which mainly includes positron-emission tomography (PET-CT), CT, MRI, or invasive pathologic assessment of cancer staging, has its limitations (23–25). There is still a clinical need for new, robust, cost-effective, and convenient, non-invasive imaging parameters to better predict the malignancy and metastasis status of solid PNs.

In recent years, deep learning has shown vast potential in medical applications and has also made great progress in pulmonary nodule diagnosis (26–34). Moreover, some researchers have predicted lymph node invasion using deep learning, radiomics models, and other methods (35–40), but predicting malignancy and M-stage metastasis for solid PNs remains inadequate. Therefore, the purpose of this study is to predict the malignancy and local or distant metastasis of solid PNs with deep learning based on chest CT images of the primary lesions. The hope is to increase the potential for the timely and reliable treatment of these highly aggressive lung nodules.

## 2. Materials and methods

### 2.1. Patients

This study was approved by the Ethics Committee of the First Affiliated Hospital of Chongqing Medical University (2022-K139), and patient confidentiality was maintained. We retrospectively reviewed the data from 1,571 consecutive patients with solid PNs who joined the management database of PNs and lung cancer in the First Affiliated Hospital of Chongqing Medical University from 1 January 2019 to 30 April 2022. The patients were divided into three groups: the benign group, the Ia-stage lung cancer group, and the T1-stage lung cancer metastasis group. The benign group can be further divided into the pathological benign group and the follow-up benign group.

**Figure 1:**
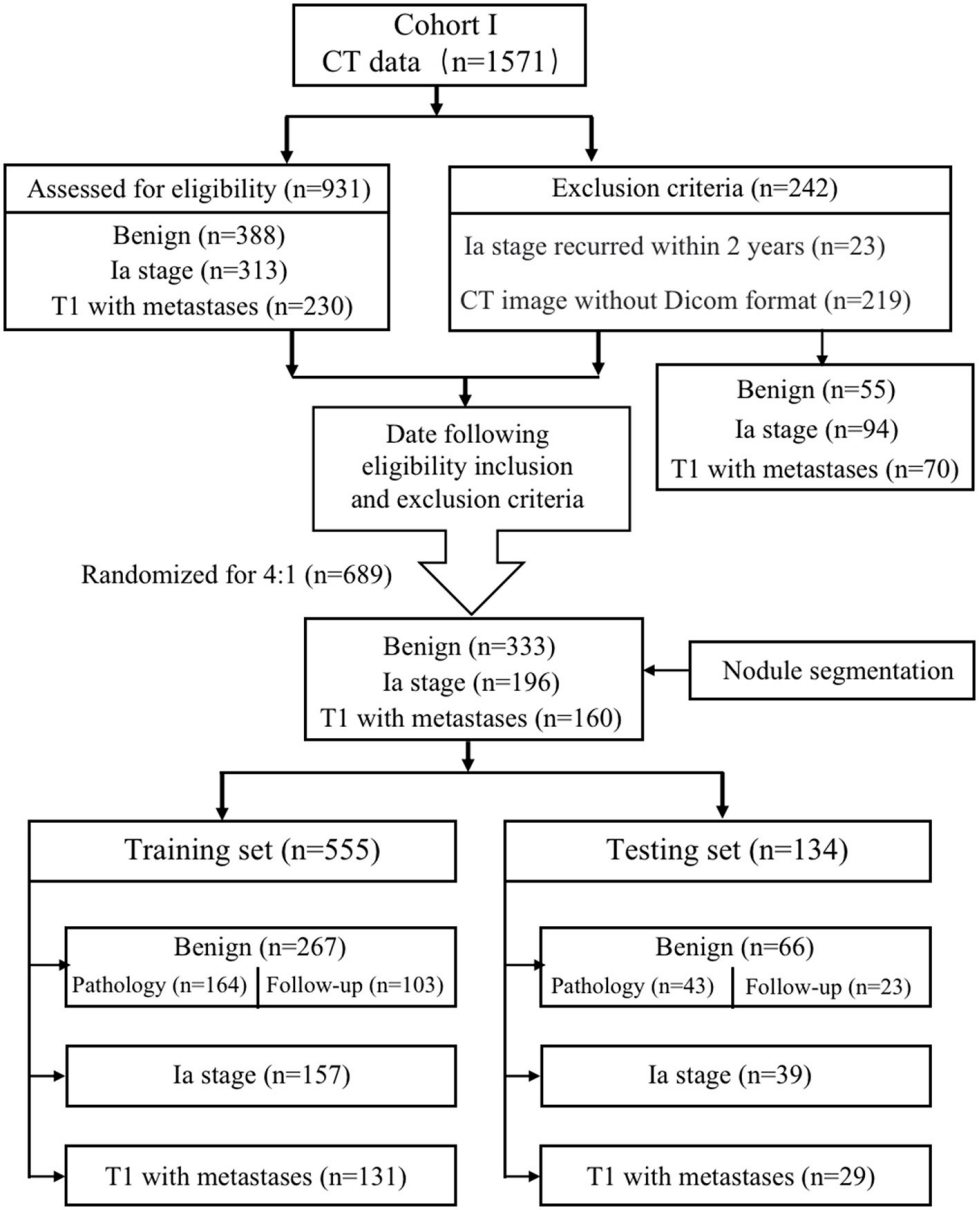
Data criteria and specification.

The inclusion criteria were as follows: dominant nodules with a size of ≤ 30.00 mm on preoperative CT images; nodule density of solid nodules; and availability of pathological report in malignant nodule patients diagnosed by non-surgical biopsy (CT-guided transthoracic biopsy and bronchoscopy non-surgical biopsy) or surgical resection. The T1-stage lung cancer metastasis patients were confirmed by PET-CT or CT combined with ultrasound and radionuclide imaging at diagnosis, imaging follow-up within 3 months, and clinicians. Pathological benign refers to getting the confirmed pathological result while excluding non-diagnostic results such as inflammation and fibroplasia that lacked follow-up data. Follow-up benign means that the solid nodules were completely absorbed, shrunk, or unchanged within 2 years of the follow-up period. The exclusion criteria were as follows: lack of thin CT images in DICOM format, metastatic cancer, and recurrence within 2 years post-operation in the Ia-stage lung cancer group. Finally, 689 patients were enrolled and divided into the training group and the testing group randomized for 8:2, which included the benign group (*n* = 333), the Ia-stage lung cancer group (*n* = 196), and the T1-stage lung cancer with metastasis group (*n* = 160) (Figure 1).

### 2.2. Data collection

**Figure 2:**
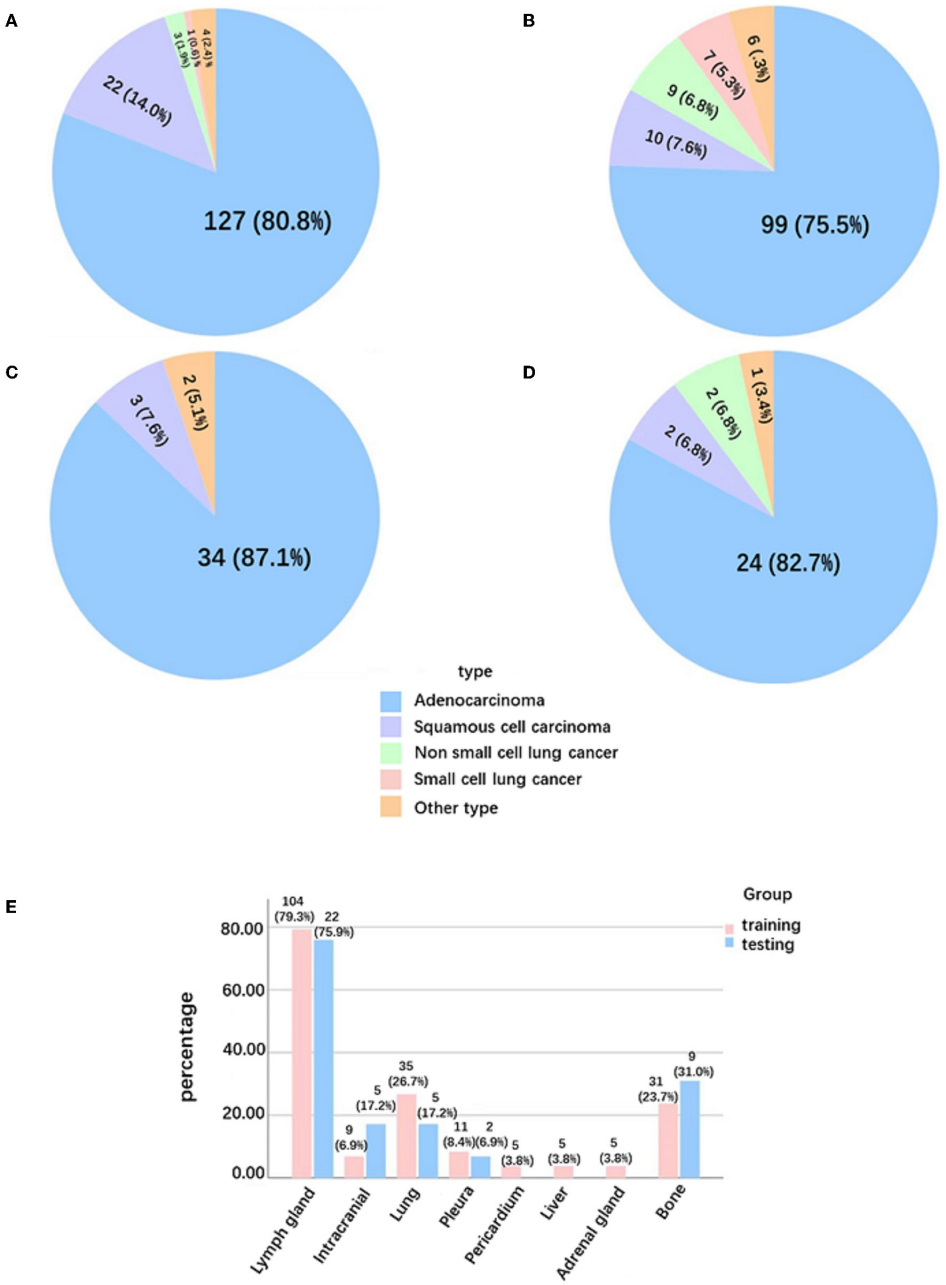
Pathological type and distribution of metastases in solid lung cancer nodules. **(A)** Pathological type of Ia-stage lung cancer patients in the training group. **(B)** Pathological type of T1-stage lung cancer patients with metastasis in the training group. **(C)** Pathological type of Ia-stage lung cancer patients in the testing group. **(D)** Pathological type of T1-stage lung cancer patients with metastasis in the testing group. **(E)** Distribution of metastases in T1-stage lung cancer patients with metastasis.

The clinical characteristics included age, gender, smoking status, history of cancer, family history of cancer, images of PNs contained size and location, histological type, lung cancer staging, distribution of metastases, and confirmed diagnosis method collected retrospectively (Table 1 and Figure 2).

**Table 1:** Baseline characteristics of the training and testing cohorts.

**Category**	**The training set**			**The testing set**			***P-*value**	***P-*value**	***P-*value**
	**Benign**	**Ia-stage**	**T1 with metastasis**	**Benign**	**Ia-stage**	**T1 with metastasis**	**Benign**	**Ia stage**	**T1 with metastasis**
Total (*n*)	267.00	157.00	131.00	66.00	39.00	29.00			
Male	121.00	88.00	79.00	27.00	18.00	19.00	0.47	0.27	0.60
Female	146.00	69.00	52.00	39.00	21.00	10.00			
Average age (years)	55.60	65.20	64.20	52.10	66.50	66.30	0.03	0.87	0.24
Smoking history (*n*, %)	94.00, 34.8%	70.00, 44.5%	69.00, 52.6%	21.00, 31.3%	15.00, 38.4%	13.00, 44.8%	0.22	0.49	0.44
History of cancer (*n*, %)	6.00, 22.4%	7.00, 4.4%	7.00, 5.3%	1.00, 1.5%	2.00, 5.1%	0.00	0.78	0.86	0.20
Family history of cancer (*n*, %)	52.00, 19.4%	12.00, 7.7%	17.00, 12.9%	12.00, 18.0%	4.00, 10.2%	1.00, 3.4%	0.75	0.59	0.14
**Dominant nodules**									
Size (mm ± SD)	12.34	16.56	19.79	13.04	16.94	20.10	0.42	0.00^*^	0.82
location (*n*, %)							0.08	0.11	0.42
RUL	72.00, 27.0%	51.00, 32.4%	40.00, 30.4%	24.00, 35.8%	15.00, 38.4%	11.00, 37.9%			
RML	31.00, 11.8%	12.00, 7.6%	9.00, 6.8%	4.00, 5.9%	7.00, 17.9%	0.00			
RLL	56.00, 20.7%	28.00, 17.8%	23.00, 17.5%	18.00, 26.8%	2.00, 5.1%	3.00, 10.3%			
LUL	48.00, 17.7%	33.00, 21.0%	42.00, 32.0%	14.00, 20.8%	6.00, 15.3%	9.00, 31.0%			
LLL	59.00, 22.5%	33.00, 21.0%	17.00, 13.0%	7.00, 10.4%	9.00, 23.0%	6.00, 20.6%			
Malignant pathological results (*n*, %)								0.497	0.733
Adenocarcinoma		127.00, 80.8%	99.00, 75.5%		34.00, 87.1%	24.00, 82.7%			
Squamous carcinoma		22.00, 14.0%	10.00, 7.6%		3.00, 7.6%	2.00, 6.8%			
Non-small cell carcinoma		3.00, 1.9%	9.00, 6.8%		0.00	2.00, 6.8%			
Small cell carcinoma		1.00, 0.6%	7.00, 5.3%		0.00	0.00			
Other		4.00, 2.4%	6.00, 4.3%		2.00, 5.1%	1.00, 3.4%			
PET-CT (*n*, %)	19.00, 7.0%	47.00, 29.9%	46.00, 35.1%	3.00, 4.5%	18.00, 46.15	13.00, 44.8%	0.45	0.05	0.37
MaxSUV value	17.20	22.40	24.10	7.40	11.20	13.10	0.94	0.22	0.81
Clinical stages (*n*, %)									0.19
Ia stage		155.00, 98.7%			39.00, 100%				
IIb stage			20.00, 15.5%			1.00, 3.4%			
III stage			47.00, 35.7%			13.00, 44.8%			
IV stage			54.00, 48.0%			15.00, 51.7%			
Diagnosis method (*n*, %)							0.0001^*^		
Pathological confirmed	159.00, 55.9%	157.00, 100%	131.00, 100%		39.00, 100%	29.00, 100%			
Follow-up confirmed	111.00, 44.1%	0.00	0.00	23.00, 34.3%	0.00	0.00			

The ^*^ symbol indicates the value of *P* < 0.05.

### 2.3. CT scanning parameters

All patients underwent chest CT scanning in our Department of Radiology before receiving a confirmed diagnosis using the following scanners: SOMATOM Perspective (Siemens Healthineers, Erlangen, Germany), SOMATOM Definition Flash (Siemens Healthineers, Erlangen, Germany), or Discovery CT750 HD (GE Healthcare, Milwaukee, WI, USA) with the following parameters: 120 kVp; 80 mAs; pitch 1.0; and collimation 0.6 mm, respectively. All imaging data were reconstructed using a medium sharp reconstruction algorithm with a thickness of ≤ 1 mm. CT scans were obtained from all patients in the supine position at full inspiration.

### 2.4. Development of the deep learning system

**Figure 3:**
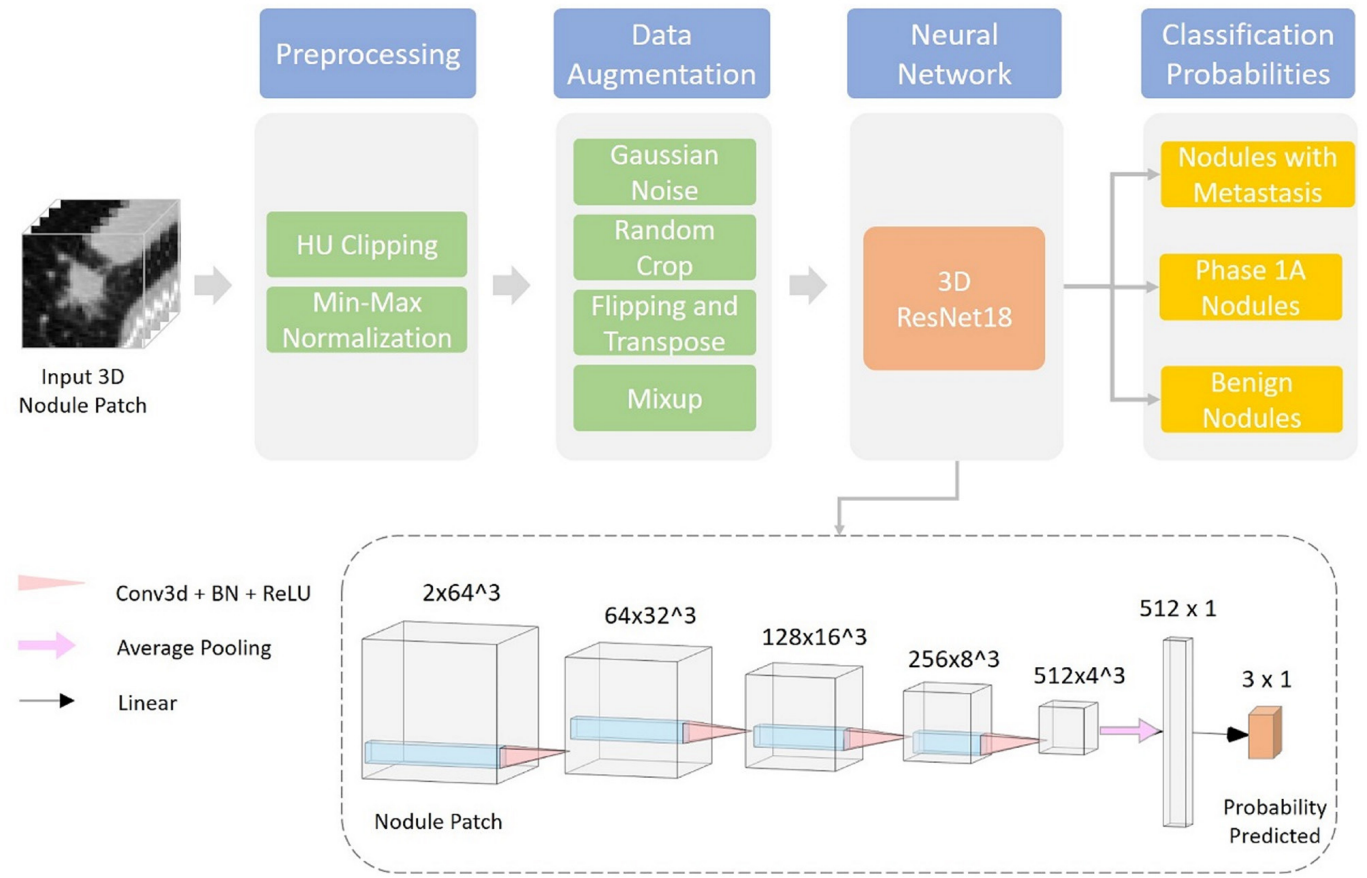
Overview of the deep learning system adopted in the diagnosis of pulmonary solid nodules.

Gives a visualization of the proposed deep learning system. This section is organized into two parts: data preprocessing and classification network (Figure 3).

### 2.5. Data preprocessing

Before being fed into the neural network, each data sample is preprocessed using the following steps:

1. Resample the CT volume *X*_*vol*_ into the spacing of 1 mm ^*^ 1 mm ^*^ 1 mm using trilinear interpolation and obtain the normalized volume *X*_*norm*<*uscore*>*vol*_;
2. Crop a 64 mm ^*^ 64 mm ^*^ 64 mm patch *X*_*norm*<*uscore*>*patch*_ around the center of each nodule from the resampled CT volume;
3. Clip HU values of *X*_*norm*<*uscore*>*patch*_ into [−1,000, 400] (equivalent to torch.clamp(x_norm_patch, −1,000, 400) or numpy.clip [x_norm_patch, −1,000, 400)];
4. Apply HU value min–max normalization, normalize HU values into [0, 1], and obtain the final output of data preprocessing $X_{final}=\frac{X_{norm<uscore>patch}-\min(X_{norm<uscore>patch})}{\max(X_{norm<uscore>patch})-\min(X_{norm<uscore>\;patch})}$.

The resampling step ensures isotropy along each dimension of the 3D nodule patch, which facilitates training of the 3D convolutional neural network. HU clipping and normalization filter out irrelevant noises in the CT patch and stabilize the training of the deep learning model.

### 2.6. Classification network

We used a 3D ResNet18 (41, 42) as the classification network in our experiments. The input of the model is a preprocessed 3D patch, together with the nodule segmentation obtained from the segmentation system developed by Dianei Technology, Shanghai in a previous study (27). The model outputs the classification probabilities of the three following probabilities: nodules with metastasis, phase 1A nodules, and benign nodules.

We train the deep learning model for 100 epochs using the AdamW (43) optimizer with a batch size of 64. The learning rate is adjusted following a cosine learning rate decay schedule (44) from 10^−3^ to 10^−6^. Hyperparameters are selected according to the network performances of 3-fold cross-validation on the training and validation datasets. The split of cross-validation is done randomly and stratified using the nodule classification labels. To alleviate overfitting caused by the limited dataset size and to improve the generalization performance of the model various data augmentation techniques were adopted during training. A full list of data augmentation is as follows:

1. Random Gaussian noise;
2. Random crop near the center;
3. Random flipping and transposing;
4. Mixup (45).

With a single forward pass and an input 3D patch of 64 mm ^*^ 64 mm ^*^ 64 mm from the CT scan, the trained network can predict the three-class probability together with the nodule mask. The nodule is classified as the category with highest probability.

### 2.7. Testing the performance of deep learning in the diagnosis of solid PNs

To test the effectiveness of our proposed method in predicting malignancy and metastasis of solid PNs, we evaluated its performances using three-class accuracy and AUC scores on predictions of nodule malignancy (benign vs. malignant+metastasis) and metastasis (benign+malignant vs. metastasis). Furthermore, we performed subgroup analysis in the following settings:

1. Total: In this setting, we evaluated our model on the entire dataset;
2. Follow-up benign: In this setting, we performed evaluations on all malignant nodules and progress-free benign nodules during follow-up visits. This setting is considered easier since the diagnosis evidence is more obvious, where we expect higher performances;
3. Pathological benign: In this setting, we included only benign nodules confirmed by pathological results and also all malignant nodules. Compared with the follow-up benign setting, this setting can be regarded as a differential diagnosis, which is more challenging for both deep learning models and human experts.

### 2.8. Observer studies

To compare the performance of the deep learning system with that of humans, an observer study of four clinicians was conducted. The specialization and years of experience of these clinicians are given in Table 2. All 134 cases in the test dataset were included in the observer studies. We evaluated the performances of both the deep learning model and clinicians using the F1 score to balance both precision and sensitivity. Meanwhile, we analyzed the inter-rater consistency among human experts in diagnosing solid nodules using Cohen's kappa scores.

**Table 2:** Specializations and years of experience of clinicians in observer studies.

	**Specialization**	**Years of experience**
Clinician A	Respiratory medicine	2
Clinician B	Respiratory medicine	5
Clinician C	Respiratory medicine	10
Clinician D	Radiology	10

### 2.9. Diagnosis accuracy of human–computer collaboration

In the observer studies, we conducted experiments to investigate whether human–computer collaboration can further enhance diagnostic accuracy. In the task of malignancy diagnosis, we combined human expert opinions with deep learning model predictions by a simple strategy, adding binary clinician diagnoses into model prediction probabilities with different weights:


$$\begin{array}{c} p_{HC}=w_{H}1_{H}+p_{C}\; \end{array}$$

Where *p*_*HC*_ is the human–computer collaboration probability, *w*_*H*_∈[0, 1] is the weight of human diagnosis, 1_*H*_ is the binary malignancy prediction from clinicians, and *p*_*C*_∈[0, 1] is the malignancy probability given by the deep learning model.

### 2.10. Statistical analysis

SPSS 25.0 software was used for statistics, and the sorted data were imported into SPSS for weighted data analysis. The independent sample *t*-test in the software analysis list was used for the *P*-value analysis of age and diameter data, and Pearson's χ2 test in the software analysis list was used for the *P*-value analysis of other data. A *P*-value of < 0.5 was defined as statistically significant.

## 3. Results

### 3.1. Clinical and pathological characteristics

A total of 333 benign nodule patients, 196 Ia-stage lung cancer patients, and 160 T1-stage lung cancer metastasis patients were enrolled. The average age was 54.82 ± 12.13 years, 65.26 ± 9.78 years, and 64.59 ± 9.48 years, respectively. The diameter of nodules was 12.53 ± 6.36 mm, 16.64 ± 5.62 mm, and 19.83 ± 5.84 mm in the benign nodules group, Ia stage group, and T1-stage metastasis groups, respectively (Appendix 1). The three groups were further divided into a training and testing group, randomized for 8:2. There was no significant difference in the clinical data between the training set and the testing set, as shown in Table 1 (*p* > 0.05). The metastases sites of T1-stage lung cancer in the training group and testing group were mainly distributed in the lymph nodes (79.3%,75.9%), lung (26.7%, 17.2%), bone (23.7%, 31%), pleura (8.4%, 6.9%), adrenal gland (3.8%, 0%), and brain (6.9%, 17.2%) (Figure 2).

### 3.2. Performance of deep learning in the diagnosis of solid PNs

Since our dataset includes both benign nodules confirmed by pathological results and those diagnosed as benign via non-progression during follow-up visits, we evaluated our deep learning model accordingly. Specifically, we reported model performances on the following three settings:

1. Total: In this setting, we evaluated our model on the entire dataset.
2. Follow-up benign: In this setting, we performed evaluations on all malignant nodules and progress-free benign nodules during follow-up visits. This setting is considered easier since the diagnostic evidence is more obvious, and we expect higher performance.
3. Pathological benign: In this setting, we included only benign nodules confirmed by pathological results and all malignant nodules. Compared with the follow-up benign setting, this setting is more challenging for both deep learning models and human experts.

Overall, our deep learning model achieved a three-class accuracy of 64.93% and AUC scores of 80.37% and 86.44% in malignancy and metastasis prediction, respectively. For the follow-up benign subset, our model reached an even higher three-class accuracy of 72.53%, a malignancy prediction AUC of 93.48%, and a metastasis prediction AUC of 87.93%. In terms of the pathological benign group, which is considered difficult to diagnose, our model achieved a decent three-class accuracy of 59.46% and scored 73.36% and 83.18% on the malignancy and metastasis prediction AUCs, respectively (Table 3).

**Table 3:** Model performances in the diagnosis of solid pulmonary nodules.

	**Accuracy**	**AUC**	
		**Malignancy**	**Metastasis**
Total	64.93%	80.37%	86.44%
Follow-up benign (*n* = 103)	72.53%	93.48%	87.93%
Pathological benign (*n* = 164)	59.46%	73.36%	83.18%

The AUC of “malignancy” denotes the model performance when classifying benign vs. malignant+metastasis, while that of metastasis denotes benign+malignant vs. metastasis.

### 3.3. Benchmarking deep learning against clinicians for malignancy prediction performance

**Figure 4:**
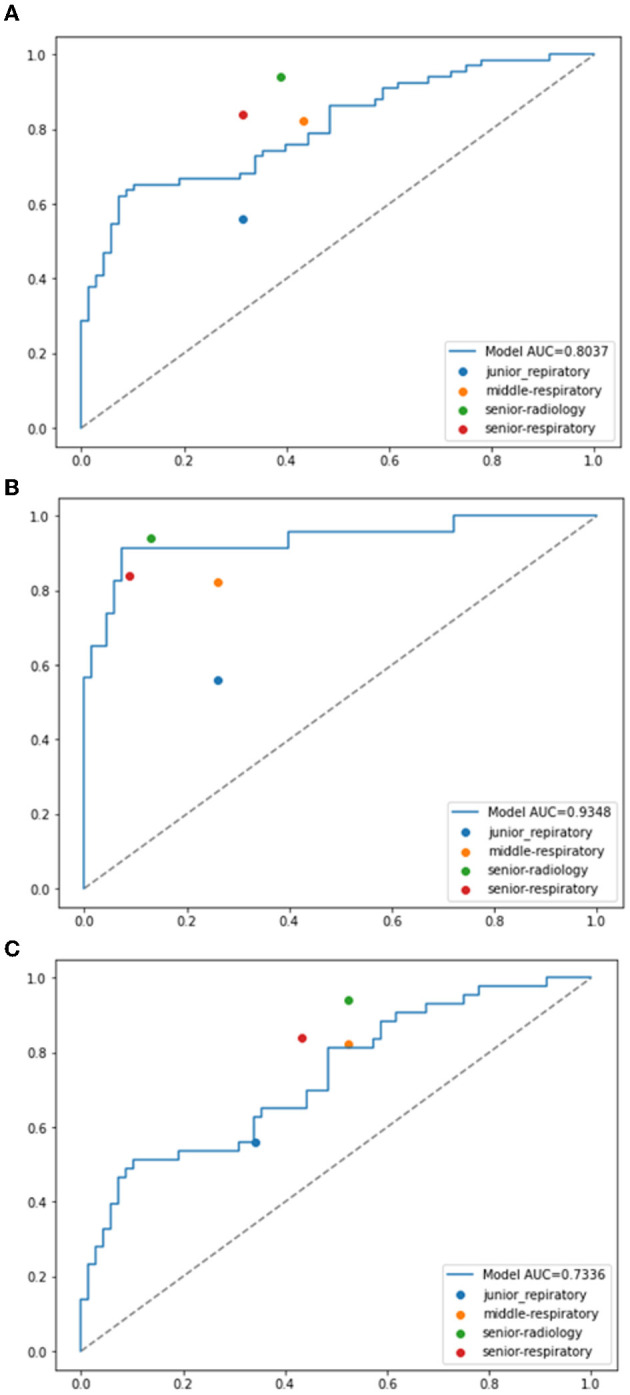
ROC curves of the proposed model compared with the performances of clinicians. **(A)** Malignancy prediction performances compared with total benign nodules data. **(B)** Malignancy prediction performances compared with the follow-up benign nodules data. **(C)** Malignancy prediction performances compared with the pathological benign nodules data.

The deep learning method outperformed the junior respiratory clinician (Clinician A) and the respiratory clinician with 5 years of experience (Clinician B) in the overall evaluation and both subgroups. Our proposed model was on par with the senior respiratory clinician (Clinician C), with slightly inferior performance on the entire dataset (77.11% vs. 78.08%) and better performances in both subgroups (93.43% vs. 89.76% in the follow-up group and 79.50% vs. 79.17% in the pathological group). Nevertheless, our proposed model fell short when compared with the senior radiologist (Clinician D), but not by a large margin. Such performances show that the deep learning model is promising when it comes to facilitating decisions similar to human clinicians in the complex task of solid nodule diagnosis (Table 4 and Figure 4). In contrast, human clinicians behave inconsistently, with a highest Cohen's kappa score of 0.4306 (Table 5). The low inter-rater consistency shows that our proposed deep learning model has better diagnostic stability in such scenarios.

**Table 4:** F1 scores of deep learning model and clinicians on the prediction of nodule malignancy.

	**Years**	**Total**	**Follow-up benign**	**Pathological benign**
Clinician A	2	59.84%	67.86%	62.81%
Clinician B	5	73.20%	86.15%	76.19%
Clinician C	10	78.08%	89.76%	79.17%
Clinician D	10	81.01%	94.81%	82.58%
3D ResNet	NA	77.11%	93.43%	79.50%

NA stands for “not applicable”.

**Table 5:** Inter-rater consistency of four clinicians in observer studies, measured by Cohen's kappa.

	**Clinician A**	**Clinician B**	**Clinician C**	**Clinician D**
Clinician A	-	0.0745	0.0192	0.2401
Clinician B	-	-	0.3286	0.2601
Clinician C	-	-	-	0.4306
Clinician D	-	-	-	-

### 3.4. Human–computer collaboration

**Figure 5:**
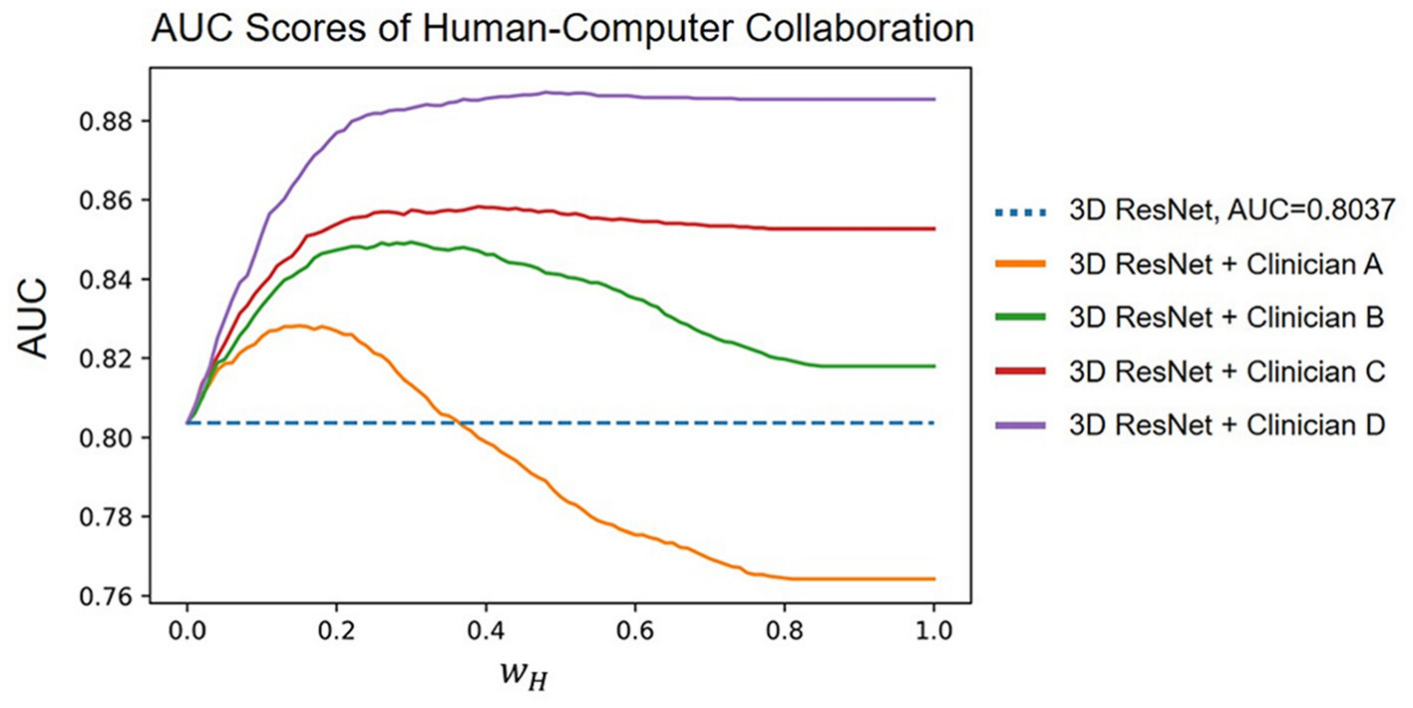
Diagnosis accuracy of human–computer collaboration.

The results of combining human and computer diagnoses with different *w*_*H*_∈[0, 1] with steps of 0.01, where *w*_*H*_ controls the weight of the human experts in collaboration. We found that Clinicians B, C, and D improved the AUC score of the deep learning model regardless of the value of *w*_*H*_. Clinician D increased the model AUC from 80.37% to 88.73% at most. Empirically, we observed that *w*_*H*_ = 0.22 improved the average AUC score the most. Under this hyperparameter setting, the model AUC is increased to 82.60%, 84.83%, 85.54%, and 88.00% when combined with Clinicians A, B, C, and D, respectively. Our human–computer collaboration experiments show that the proposed model becomes more accurate when working with humans, demonstrating its great potential in clinical practice (Figure 5).

## 4. Discussion

The best clinical management of PNs requires the evaluation of the probability of malignancy, which determines the most cost-effective diagnostic and therapeutic strategies. Previous studies on the diagnosis of solid PNs mainly focused on using radiomics models or nomograms or included only pathologically benign nodules (14, 15, 29) and did not take advantage of the deep learning technique. Heuvelmans et al. trained and validated a lung cancer prediction convolutional neural network on an independent dataset of small-to-intermediate nodules sized 5–15 mm and demonstrated its excellent performance in identifying benign nodules (46). In their research, benign nodules were determined by screenings and follow-ups until 7 years after baseline in the National Lung Screening Trial as well as solid nodules (46). Moreover, larger benign solid PNs are usually characterized by overlapping imaging features and are easily misdiagnosed and overtreated. Our study provided evidence that deep learning methods based on CT images of the primary lesions can be used to predict the malignancy of solid PNs (size ≤ 30 mm) and performed better than two junior or middle-level clinicians, only slightly inferior to the senior radiologist. In the follow-up benign subset, our model reached an even higher three-class accuracy of 72.53% and a malignancy prediction AUC of 93.48%. In terms of the pathological benign group, which is considered difficult to diagnose, our model achieved a decent three-class accuracy of 59.46% and scored 73.36% on the malignancy prediction AUC. Our human–computer collaboration experiments show that the proposed model becomes more accurate when working with humans, demonstrating its great potential when used in clinical practice. Therefore, the proposed deep learning method can accurately diagnose solid PNs, even if they are indeterminate solid lung nodules, and has demonstrated improved performance upon working in tandem with human experts.

At present, it is difficult to detect and predict the metastasis of T1-stage lung cancer until it has already developed to a certain stage (25), but it is critical to match patients with appropriate individualized therapy strategies and predicting prognoses. Numerous studies have reported using radiomics features, deep learning, or other methods to predict lymph node metastasis, but not the M staging of lung cancer. Beck et al. reported that the deep cubical nodule transfer learning (CUBIT) algorithm, using transfer learning and a 3D convolutional neural network (CNN) based on CT scan images, can accurately predict LVI or nodal involvement in primary non-small cell lung cancer (NSCLC) (36). Nie et al. reported that a radiomics nomogram incorporating the Rad-score and clinical and PET/CT parameters shows favorable predictive efficacy for lymph vascular invasion status in lung adenocarcinoma (47). Zhang et al. established a PET/CT nomogram based on the metabolic information (SUVmax) and structural information (radiomics features) of lymph nodes for preoperative quantitative estimation of lymph node metastasis (48). Tau et al. used convolutional neural networks to predict the nodal and distant metastatic potential of newly diagnosed NSCLC on FDG PET images (49), but the authors did not specifically identify solid PNs. Tian et al. reported that the radiomics features of pretherapy CT images may be used as predictors of distant metastasis, but there were only 43 cases of solid lung cancer nodules, and only three patients had metastases in their study (25). In this research, we collected a cohort of 689 patients with solid PNs and trained a 3D CNN to predict the local or distant metastasis of nodules. On a held-out testing set of 134 cases, the deep learning approach achieved an AUC score of 86.44% for metastasis prediction. The method employed in this study can be used to predict or diagnose the metastasis of T1-stage lung cancer nodules based on CT imaging. When we are able to better evaluate the characteristics of these nodules, clinicians will have a greater chance of identifying highly aggressive lung cancer at its earliest stages, making treatment planning and patient stratification viable for everyone.

## 5. Conclusion

Although this proposed model shows great promise and is able to compete with senior clinicians in the solid nodule diagnosis task, there are limitations worth mentioning. First, not all patients in the metastasis group had pathological results for the metastatic sites. Because most of them were local or late-stage lung cancer patients, metastasis was mainly confirmed by non-invasive systemic screening, clinician experience, or follow-up in ethics. Second, this was a single-center retrospective study with a relatively small sample size. However, because of the difficulty of medical data collection, it is the largest sample size reported in T1-stage solid lung cancer patients with metastases, according to the literature. Multicenter studies with larger datasets can be validated in the future. In addition, only one single CT scan is included for each patient in our experiments, while in practice clinicians usually take multiple follow-up CT scans into account. The next step is to design a prospective study in which follow-up CT sequences can be added to make the best use of information from multiple time points and to improve diagnostic accuracy (50, 51). Additionally, our human–computer collaboration experiment settings are not close enough to real-world clinical settings. This is due to the labor intensiveness of having all four clinicians carry out the diagnosis once again. In our future research, we will experiment with human experts diagnosing with computer assistance in real-world scenarios. However, we argue that our human–computer collaboration method has its own benefit since it draws a frontier of possible collaboration results, demonstrating that human–computer collaboration is a bonus under various levels of human trust. Our approach is also robust against human variance, which is high, as shown in our inter-rater consistency analysis. Finally, the current method focuses on modeling only the CT modality, whereas ideally, clinicians use a variety of information, such as smoking history and multiomics information (52, 53) to better estimate the metastasis and malignancy of solid PNs. Aggregating such information in our modeling may further boost its diagnostic performance.

In summary, this study provided evidence that the proposed deep learning method extracted from CT images of primary lesions can accurately diagnose the malignancy of solid PNs and its performance improves when collaborating with human experts. To the best of our knowledge, this is the first study to use deep learning with pretherapy CT images of primary tumors to judge N and M staging in T1 solid lung cancer nodules, which could help to provide optimal care for these patients. The prediction of metastasis in T1-stage lung cancer using CT images has become simple yet accurate through deep learning methods.

## Data availability statement

The datasets presented in this article are not readily available because of ethical and copyright restrictions. Requests to access the datasets should be directed to the corresponding author.

## Ethics statement

The studies involving human participants were reviewed and approved by the Ethics Committee of the First Affiliated Hospital of Chongqing Medical University. Written informed consent to participate in this study was provided by the participants' legal guardian/next of kin.

## Author contributions

LY, JY, and SG designed this study. JM, MA, WL, ZO, and JH collected the data. LY and HD performed the statistical analysis. JY, KK, and JL developed the deep learning model. LY, KK, JL, JY, and JM wrote the manuscript. LY, MA, and HD performed the procedures. All authors read and approved the final version of the manuscript.

## Appendix

**Appendix 1:** Characteristics of age and nodules size in the benign nodules group, the Ia-stage lung cancer group, and the T1-stage lung cancer with metastasis groups.

**Category**	**Benign**	**Ia-stage**	**T1 with metastasis**	**Total**
Average age (years)	54.82 ± 12.13	65.26 ± 9.78	64.59 ± 9.48	60.06 ± 12.03
Max	83	88	85	88
Min	21	32	39	21
Dominant nodules size (mm ± SD)	12.53 ± 6.36	16.64 ± 5.62	19.83 ± 5.84	15.39 ± 6.72
